# CES3 promotes NSCLC progression via lipid metabolic reprogramming regulated by TFAP2A

**DOI:** 10.7150/jca.118395

**Published:** 2026-01-01

**Authors:** Pengfei Luo, Zirui Huang, Sijuan Ding, Zhangwen Tang, Yanhong Wei, Shaohui Jiang, Ruoting Tang, Fang Li, Han Yang, Lujun Zhao

**Affiliations:** 1 Department of Radiation Oncology, Key Laboratory of Cancer Prevention and Therapy, Tianjin Medical University Cancer Institute & Hospital, National Clinical Research Center for Cancer, Tianjin's Clinical Research Center for Cancer, Tianjin 300060, China.; 2 Department of Oncology, The Central Hospital of Yongzhou, No. 396 Yiyun Road, Yongzhou City, 425000 Hunan Province, China. Hunan Provincial Demonstration Base for Medical Technology in Tumor Radiotherapy.; 3 State Key Laboratory of Oncology in South China, Guangdong Provincial Clinical Research Center for Cancer, Sun Yat-sen University Cancer Center, Guangzhou, 510060, China.

**Keywords:** non-small cell lung cancer, lipid metabolism, carboxylesterase 3, transcription factor AP-2α, proliferation

## Abstract

Metabolic reprogramming is an important feature in non-small cell lung cancer (NSCLC) that can result in therapeutic resistance. Exploring dysregulated lipid metabolism in NSCLC will accelerate the development of potential lipid biomarkers to target and control the malignant progression of NSCLC. In this study, RNA next-generation sequencing of 25 paired NSCLC specimens and adjacent normal tissues was used to find that carboxylesterase 3 (CES3) was upregulated in NSCLC. Knockdown of CES3 significantly inhibited NSCLC cell proliferation and invasion. Additionally, CES3 inhibition promoted lipid accumulation in NSCLC cells. Furthermore, we found transcription factor AP-2α (TFAP2A) could regulate CES3 levels in NSCLC. TFAP2A was found upregulated in NSCLC and correlated with poorer outcome. Inhibiting TFAP2A resulted in suppressed cell proliferation as well as invasion while increasing the lipid accumulation in NSCLC. CES3 overexpression could reverse the impact of TFAP2A inhibition on NSCLC progression. In summary, TFAP2A dysregulation resulted in CES3 overexpression and the following NSCLC tumorigenesis. Targeting the TFAP2A/CES3 axis may represent a promising therapeutic strategy for NSCLC in the future.

## Introduction

According to the most recent data from Cancer Statistics, 2022, lung cancer remains one of the leading causes of cancer-related mortality worldwide, with non-small cell lung cancer (NSCLC) accounting for over 80% of lung cancer cases 1, 2. NSCLC is a tumor with highly heterogeneous molecular profile that produces a variety of metabolic phenotypes, and metabolic reprogramming is an important feature of NSCLC that can lead to therapeutic resistance 3-5. Surgery remains the cornerstone treatment for patients with early-stage non-small cell lung cancer 6. Targeted therapies have significantly transformed the treatment landscape of NSCLC, particularly in metastatic cases, and recent research efforts have increasingly focused on their integration into earlier phases of treatment, such as neoadjuvant and adjuvant contexts 7, 8. Meanwhile, there is growing recognition of the critical role that dysregulated lipid metabolism plays in the development and progression of NSCLC 9. A growing body of research suggests that disorder of lipid metabolism is an important character in lung cancer 10. In NSCLC, lipid metabolism is significantly dysregulated, which can affect tumor cell proliferation, metastasis, and therapeutic sensitivity, ultimately leading to poor survival outcomes for patients 11. Modulation of lipid metabolic processes in NSCLC may be a therapeutic option to stop NSCLC progression 12. However, the contribution of aberrant lipid metabolism in NSCLC and the mechanisms involved remain unclear. Investigating lipid metabolism dysregulation of NSCLC will accelerate the development of potential lipid biomarkers to target with the aim of controlling the malignant progression of NSCLC 13. In summary, studying the molecular mechanisms of lipid metabolism remodeling in NSCLC will contribute to better understanding of the molecular targets and pathways to provide novel theoretical basis to treat NSCLC 14.

Lipid metabolism is considered an important player in various tumor biological processes 15, 16. Carboxylesterase 3 (CES3) participates in multiple lipid metabolic processes, including the hydrolysis of triglycerides and the assembly of very low-density lipoproteins (VLDL) 17. Studies have shown that deletion of CES3 reduces plasma triglyceride (TG), fatty acid, and cholesterol levels, improves insulin sensitivity, and reduces tumor weight in tumor-bearing mice 18. CES3 is aberrantly expressed in a variety of tumors, He *et al.* 19 demonstrated that CES3 expression is significantly downregulated at both mRNA and protein levels in colonic adenocarcinoma compared to normal tissues. They found that higher CES3 expression is associated with better patient prognosis and improved immunotherapy efficacy, likely through its relationship with tumor-infiltrating immune cells. In hepatocellular carcinoma, Dong *et al.* 20 identified CES3 as one of several genes inversely correlated with the glycolytic phenotype characteristic of tumor metabolism, with decreased expression linked to poor outcomes. Their study also revealed that Angiotensin-Converting Enzyme 2 (ACE2), functioning within this metabolic network alongside CES3, suppresses tumor progression by regulating metabolic and oxidative stress pathways. Quiroga *et al.* 21 reported that CES3, also known as triacylglycerol hydrolase, is downregulated early during liver cancer development in animal models, contributing to metabolic shifts that promote tumorigenesis. Notably, dietary supplementation restoring CES3 expression reduced the number and size of preneoplastic hepatic foci, indicating a preventive role for CES3 in liver carcinogenesis. Collectively, these studies highlight CES3 as a potential biomarker and therapeutic target involved in modulating tumor metabolism, immune response, and progression across multiple malignancies. However, its clinical significance and potential biological roles in NSCLC are not clear. Therefore, in-depth elucidation of the biological roles and mechanisms of CES3 in NSCLC may provide a new theoretical basis for the clinical application of its targeted therapy.

Here, we found that CES3 was upregulated in NSCLC and inhibition of CES3 resulted in suppressed cell proliferation as well as invasion. Besides, inhibiting CES3 enhanced lipid accumulation of NSCLC cells. Furthermore, we found transcription factor AP-2α (TFAP2A) could regulate CES3 expression in NSCLC. In summary, TFAP2A dysregulation resulted in CES3 overexpression and progression of NSCLC. We could use TFAP2A/CES3 as a novel strategy to treat NSCLC in the future.

## Methods

### Data collection

TFAP2A expression in lung adenocarcinoma (LUAD) cohort including 59 normal samples and 526 cancer samples as well as the lung squamous cell carcinoma (LUSC) cohort including 49 normal samples and 501 cancer samples, were downloaded from ENCORI/starbase project (https://rnasysu.com/encori/panCancer.php). The survival analysis for TFAP2A in LUAD was also downloaded from ENCORI/starbase project (https://rnasysu.com/encori/panCancer.php), patients in the LUAD cohort were stratified into two groups based on TFAP2A expression levels: those with expression above the cohort median were categorized as the high TFAP2A expression group, while those with expression below the median were designated as the low TFAP2A expression group.

### Tissue specimens

25 NSCLC tissues (T) along with paired normal tissues (N) were collected and submitted to RNA next-generation sequencing. Moreover, 33 paired NSCLC tissues along with adjacent normal tissues were collected and submitted to qRT-PCR assays. This study was approved by Sun Yat-sen University Cancer Center. Informed consent forms were signed individually.

### Cell lines and transfection

Human normal lung cell line (Beas2b) and human NSCLC cell lines (PC9, H1299, H1975 and A549) were bought from ATCC (USA) and cultured with RPMI-1640. PC9 and A549 were treated with TFAP2A siRNAs, CES3 overexpression vector or control 22. These cell lines were cultured under standard conditions at 37°C in a humidified atmosphere containing 5% CO₂. Supplementary Material Table S1 showed TFAP2A siRNA sequences and Table S2 showed primer sequences for qRT-PCR.

### CCK-8 assay

3 × 10^3^ PC9 and A549 cells were treated by WWL229 23, 24 (CES3-specific inhibitor, MCE, #1338575-28-2, USA, 2 μmol/L) or transfected by siRNAs against TFAP2A with or without CES3 overexpression vector. After 48 h, CCK-8 reagent was added and 450 nm absorbance was collected after an hour.

### Colony formation assay

1 × 10^3^ PC9 and A549 cells were treated by WWL229 or transfected by siRNAs against TFAP2A with or without CES3 overexpression vector. After 14 days, the colonies were fixed, stained and counted 25.

### Transwell assay

In this study, PC9 and A549 cells were utilized at a density of 3 × 10^4^ cells per well. Cell suspensions were seeded into the upper chambers of Transwell inserts pre-coated with Matrigel to simulate the extracellular matrix. The lower chambers were filled with culture medium containing 20% serum to serve as a chemoattractant. Following incubation at standard conditions for several hours up to 24 hours, cells remaining on the upper surface of the membrane that did not invade were carefully removed. Invaded cells were fixed, stained, and subsequently quantified under a microscope to assess the invasive potential of the cells.

### Oil red O staining

PC9 and A549 cells were seeded then treated by WWL229 or transfected by siRNAs against TFAP2A with or without CES3 overexpression vector. Then, cells were subsequently fixed and stained with Oil Red O reagent, followed by imaging under a light microscope.

### Western blotting

RIPA lysis buffer and PMSF were used to isolate proteins and 10% SDS-PAGE was used to separate the proteins. Next, the proteins were transferred onto PVDF membranes. The following antibodies were used to incubate the membranes: TFAP2A (1:1000, #ab108311, abcam), CES3 (1:1000, #DF12580, Affi8nity), FABP4 (1:1100, #DF6035, Affinity), ATGL (1:1200, #DF7756, Affinity), and GAPDH (1:12000, #AF7021, Affinity) 26.

### Statistical analysis

Data analysis was performed with SPSS 27.0 software. The analysis of group comparisons involved the utilization of t-tests and one-way ANOVA. Data is shown as mean ± standard deviation. When *P* < 0.05, the statistical significance was established.

## Results

### CES3 is elevated in NSCLC

**Figure 1:**
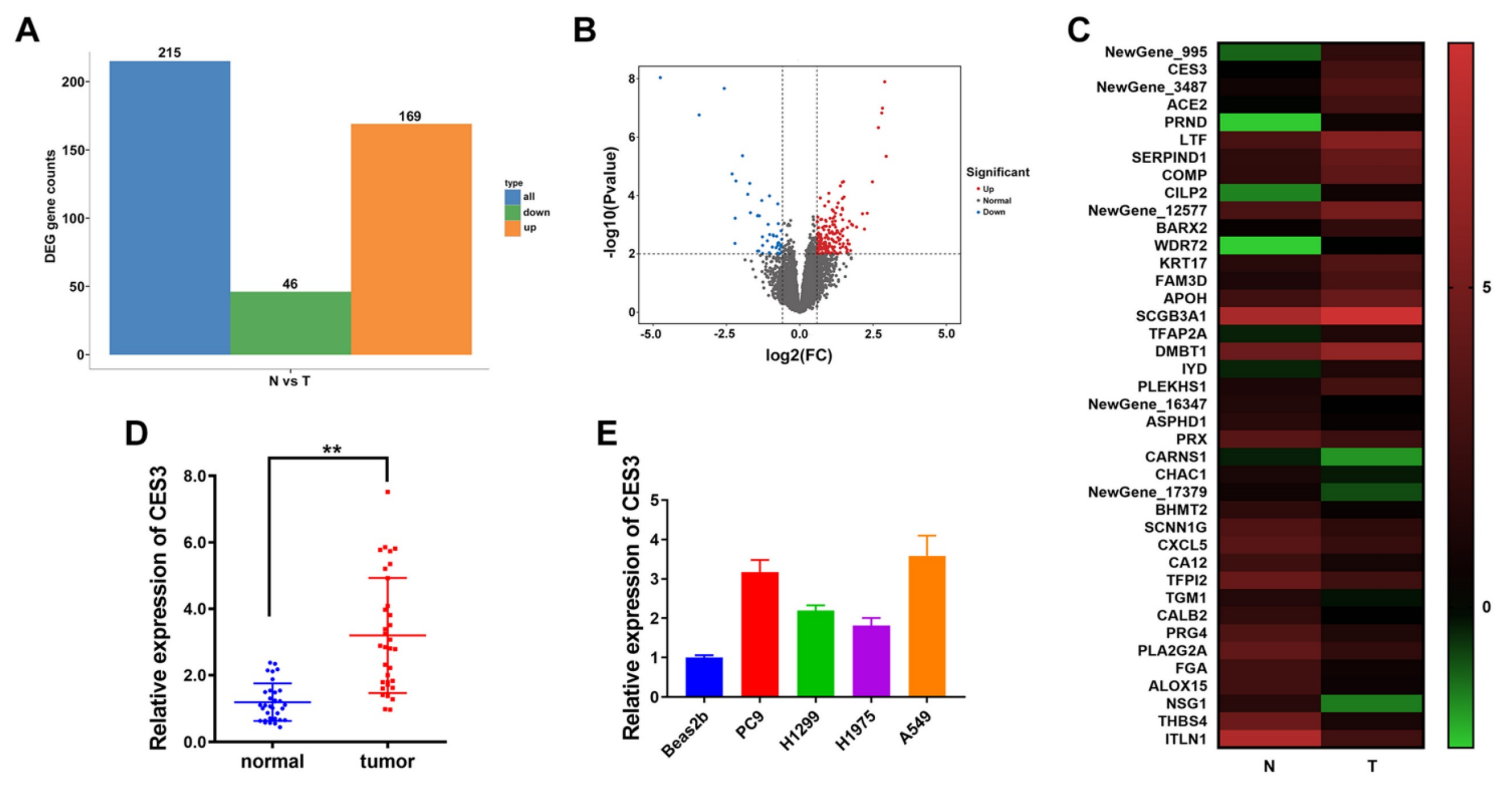
** CES3 is elevated in NSCLC. (A)** Differentially expressed genes in 25 paired NSCLC samples (T) and adjacent normal samples (N). Orange: up regulated; Green: down regulated. Fold Change ≥ 2 and *P* ≤ 0.01. **(B)** Volcano plots showing genes differentially expressed in 25 paired NSCLC samples (T) and adjacent normal samples (N). Red: upregulated; Blue: downregulated. Fold Change ≥ 2 and *P* ≤ 0.01. **(C)** Hierarchical clustering generated by GraphPad prism 9.0 with the Log2(FPKM) of DEGs showing the top 20 upregulated or downregulated genes in 25 paired NSCLC samples (T) and adjacent normal samples (N). Red: up regulated; Green: down regulated. Color bar: Log2(FPKM) of DEGs. **(D)** CES3 levels in 33 paired NSCLC samples (tumor) and adjacent normal samples (normal) were detected by qRT-PCR. **(E)** CES3 levels in NSCLC cell lines were detected by qRT-PCR. * < 0.05, **P < 0.01.

To investigate key genes involved in the progression of NSCLC, we performed RNA sequencing using next-generation sequencing technology on 25 pairs of NSCLC tumor tissues(T) and matched adjacent normal tissues(N). The results in Figure 1A and 1B showed that 169 genes were increased and 46 genes were decreased in NSCLC. Among the top 20 upregulated genes in NSCLC, we chose CES3 for further study, whose levels were notably elevated in NSCLC (Figure 1C). Furthermore, we confirmed the elevated expression of CES in both NSCLC tissues and cell lines (Figure 1DE).

### CES3 inhibition suppresses NSCLC proliferation and invasion

**Figure 2:**
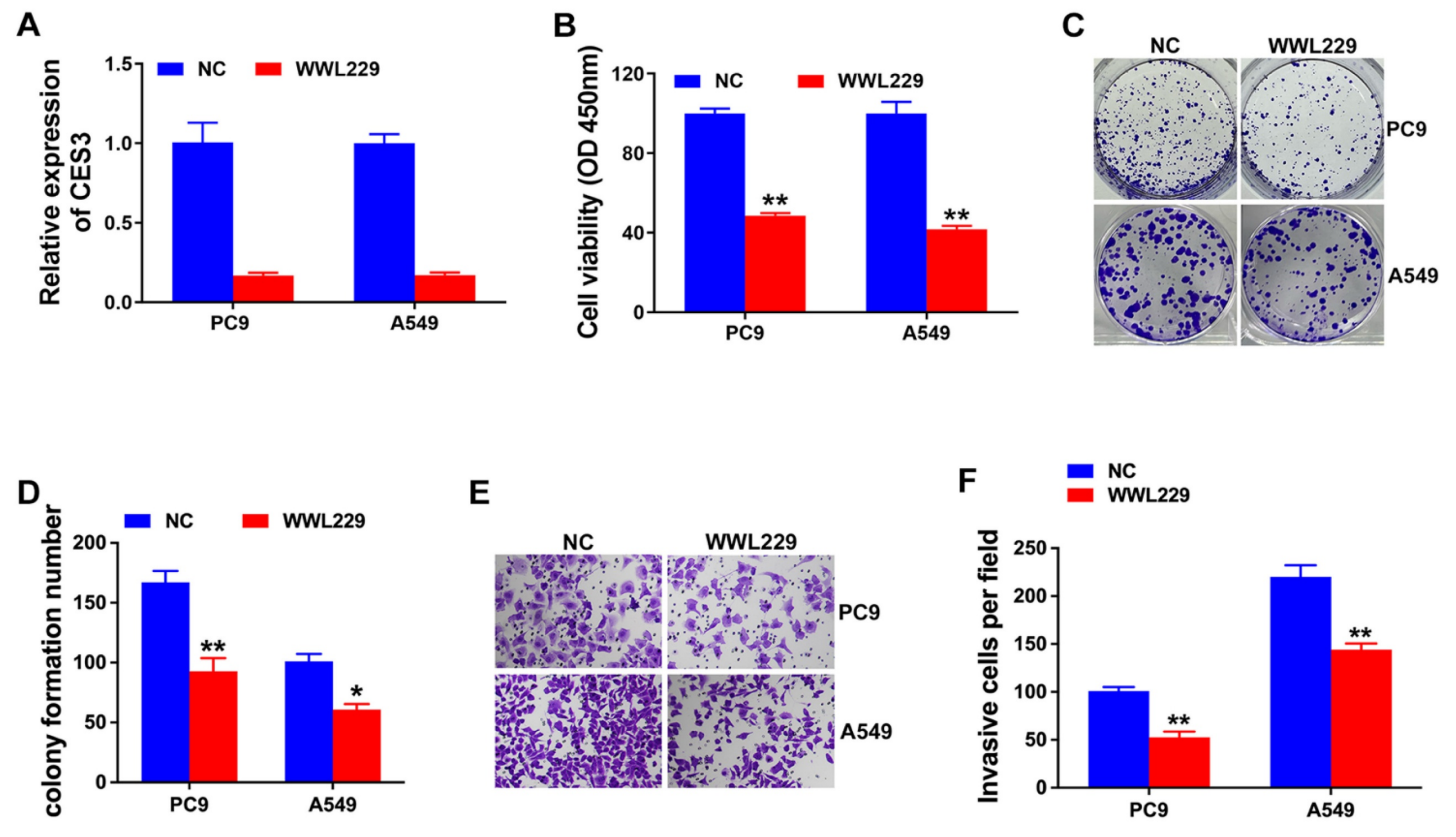
** CES3 inhibition suppresses NSCLC proliferation and invasion. (A)** CES3-specific inhibitor WWL229 was used to knock down the levels of CES3 in NSCLC cell lines. **(B)** CCK-8 assay was conducted in NSCLC cell lines after treatment with WWL229.** (C)** Represent image of colony formation assay of NSCLC cell lines after treatment with WWL229. **(D)** Colony formation number was quantified by ImageJ software. **(E)** Represent image of Transwell assay of NSCLC cell lines after treatment with WWL229. **(F)** Invasive cell number was quantified. *P < 0.05, **P < 0.01.

WWL229 is a selective inhibitor of the carboxylesterase protein CES3^27^. To investigate the role of CES3 in NSCLC, we treated the NSCLC cell lines PC9 and A549 with WWL229. qRT-PCR analysis confirmed that WWL229 effectively downregulated CES3 expression in these cells (Figure 2A). Functional assays demonstrated that CES3 inhibition significantly reduced the proliferative capacity of both PC9 and A549 cells, as shown by CCK-8 assays (Figure 2B). Additionally, colony formation assays revealed a substantial decrease in the number of NSCLC cell colonies following CES3 suppression, consistent with the reduced proliferation observed (Figures 2C and 2D). Moreover, transwell assays indicated that WWL229 impaired the invasive ability of NSCLC cells (Figures 2E and 2F), suggesting that CES3 plays a critical role in both proliferation and invasion of NSCLC cells.

### CES3 inhibition reduces NSCLC progression via lipid metabolism regulation

**Figure 3:**
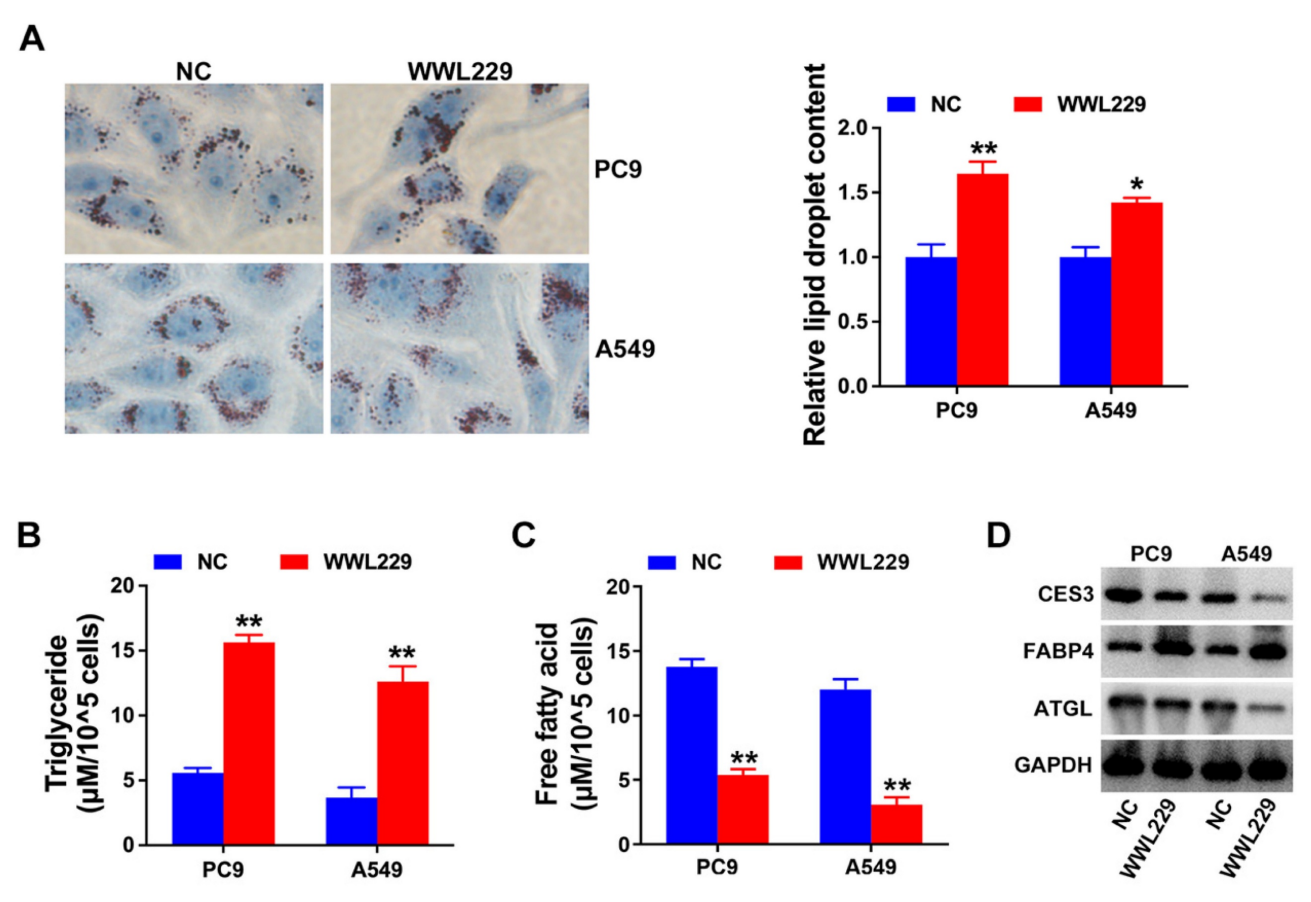
** CES3 inhibition reduces NSCLC progression via lipid metabolism regulation. (A)** Oil red O staining conducted to detect intracellular lipid contents in NSCLC cell lines after treatment with WWL229 (left). Intracellular lipid droplet contents were quantified by ImageJ software (right).** (B)** The intracellular TG levels were detected in NSCLC cell lines after treatment with WWL229. **(C)** The intracellular FFA levels were detected in NSCLC cell lines after treatment with WWL229.** (D)** Lipid metabolism related genes expressions were determined by Western blotting in NSCLC cell lines after treatment with WWL229. *P < 0.05, **P < 0.01.

Next, we explore the role of CES3 in NSCLC lipid metabolism. Oil Red O staining assay revealed that WWL229 enhanced lipid accumulation of NSCLC cells (Figure 3A). Besides, WWL229 increased NSCLC cell TG levels and reduced FFA levels (Figure 3B and 3C). Finally, western blotting assay revealed that WWL229 enhanced lipid biosynthesis related gene FABP4 expression and suppressed lipolysis related gene ATGL expression in NSCLC (Figure 3D), indicating that CES3 was an important player in NSCLC lipid metabolism.

### TFAP2A regulates CES3 expression in NSCLC

**Figure 4:**
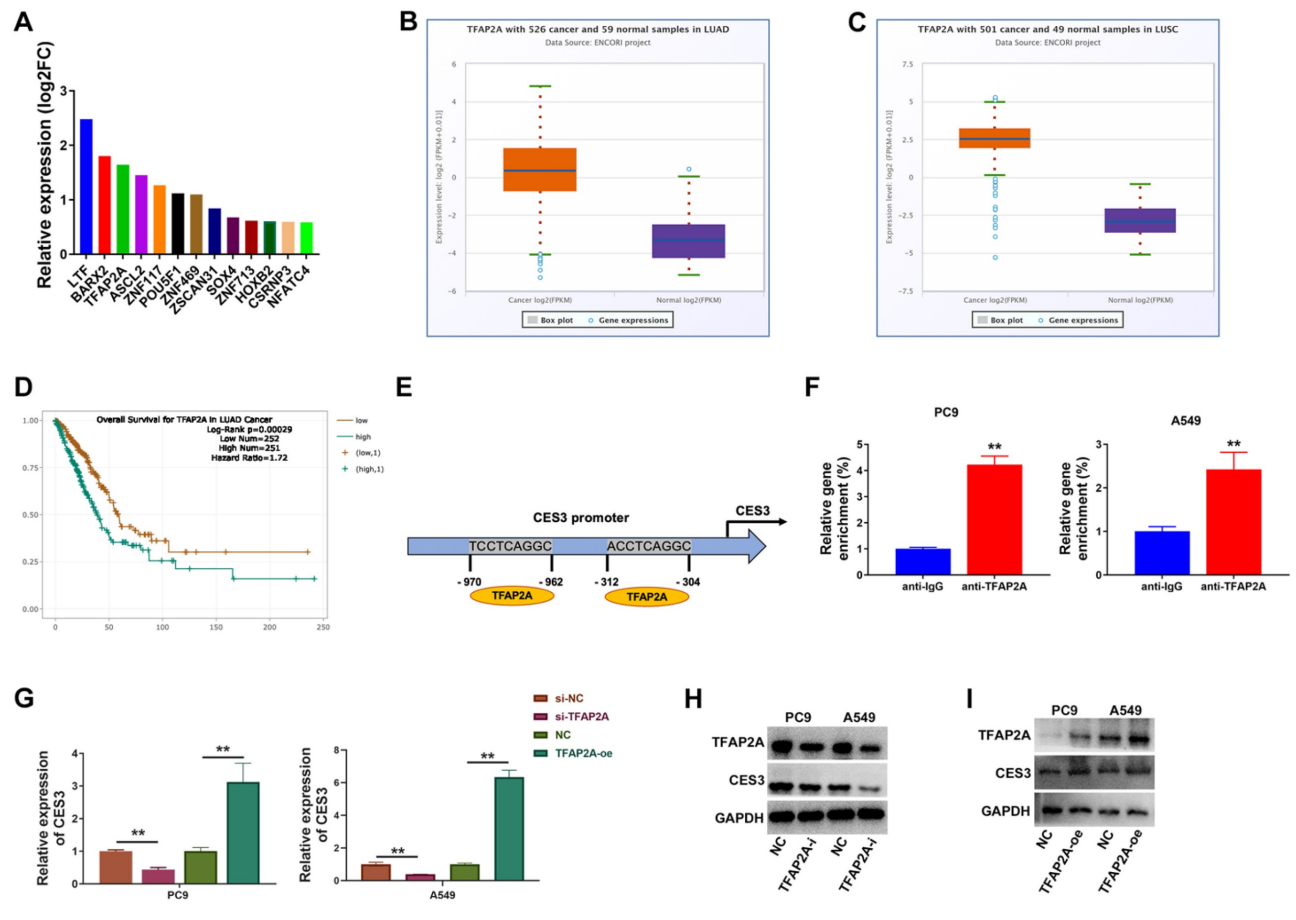
** TFAP2A regulates CES3 expression in NSCLC. (A)** Top 13 highly expressed transcription factors in NSCLC specimens.** (B)** The expression of TFAP2A in LUAD cohort including 59 normal samples and 526 cancer samples was shown.** (C)** The expression of TFAP2A in LUSC cohort including 49 normal samples and 501 cancer samples was shown.** (D)** The survival analysis for TFAP2A in LUAD was shown. **(E)** Represent diagram of the putative binding sites for TFAP2A in CES3 promoter region. **(F)** ChIP assay showing the binding of TFAP2A and CES3 promoter region.** (G)** qRT-PCR showed the regulation of TFAP2A on CES3 expression. oe: over expression.** (H)** The protein levels of TFAP2A and CES3 were detected by western blotting after inhibition of TFAP2A. **(I)** The protein levels of TFAP2A and CES3 were detected by western blotting after overexpression of TFAP2A. * < 0.05, **P < 0.01.

Next, we continued to investigate the mechanism of CES3 overexpression in NSCLC. The RNA next-generation sequencing results revealed TFAP2A as an upregulated transcription factor in NSCLC tissues (Figure 4A). Analysis of lung adenocarcinoma and lung squamous cell carcinoma cohorts from the TCGA public database revealed that TFAP2A is significantly overexpressed in tumor tissues, consistent with our experimental findings (Figures 4B and 4C). Survival analysis demonstrated that elevated TFAP2A expression correlates with poorer overall survival, suggesting its potential as a prognostic marker in non-small cell lung cancer (Figure 4D). To investigate the molecular mechanisms of TFAP2A, bioinformatic analysis predicted TFAP2A binding sites within the CES3 gene promoter (Figure 4E). Chromatin immunoprecipitation (ChIP) assays experimentally confirmed the direct binding of TFAP2A to the CES3 promoter in NSCLC cells. The enrichment of the CES3 promoter region was significantly greater in samples immunoprecipitated with the anti-TFAP2A antibody compared to the anti-IgG control. Specifically, relative gene enrichment increased more than fourfold in PC9 cells (*P* < 0.01) and over twofold in A549 (*P* < 0.01) cells, demonstrating strong and specific binding of TFAP2A to the CES3 promoter (Figure 4F). Besides, overexpression of TFAP2A could increase CES3 levels, and inhibition of TFAP2A could decrease CES3 levels (Figure 4G - 4I). Thus, we concluded that TFAP2A promoted CES3 expression in NSCLC by binding to CES3 promoter, that dysregulation of TFAP2A resulted in CES3 overexpression in NSCLC.

### TFAP2A is elevated in NSCLC and TFAP2A suppression inhibits NSCLC growth and invasion

**Figure 5:**
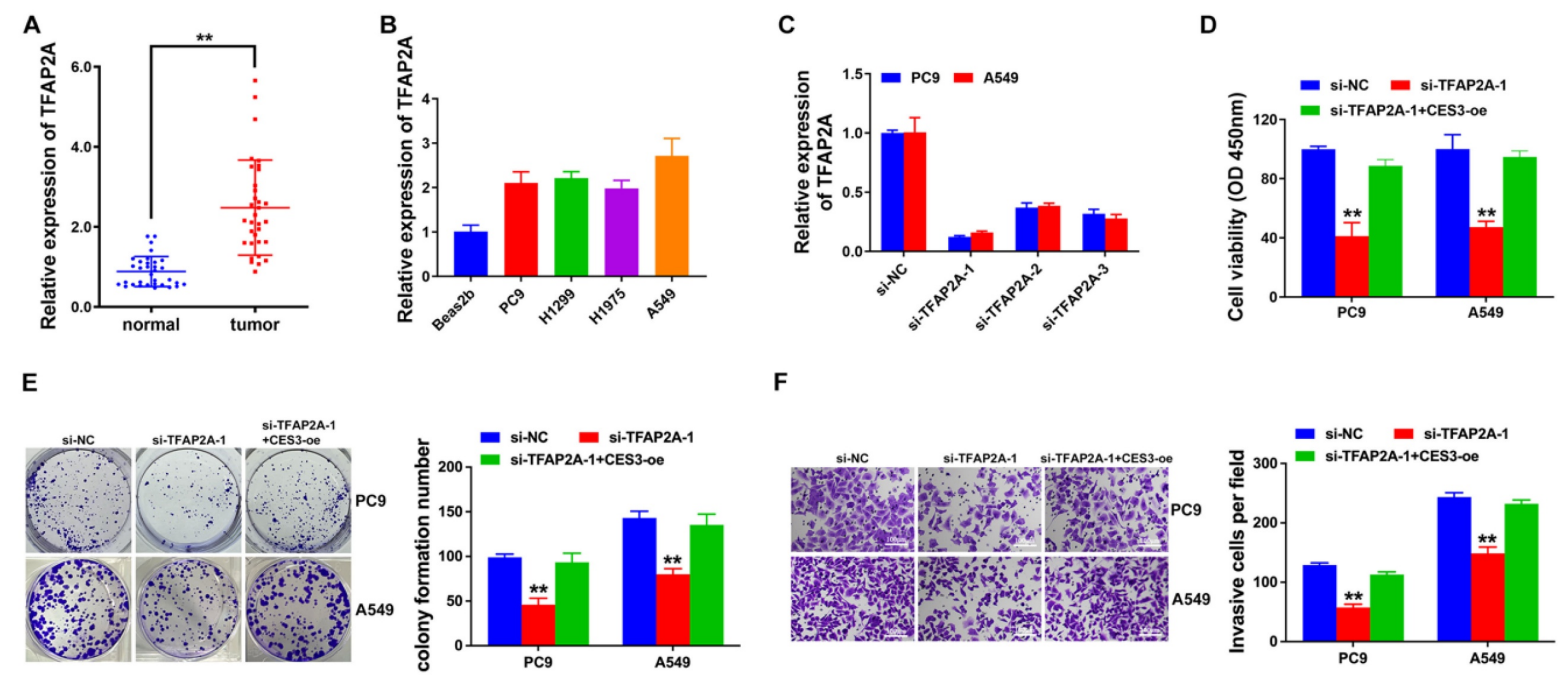
** TFAP2A is elevated in NSCLC and TFAP2A suppression inhibits NSCLC growth and invasion. (A)** TFAP2A levels in 33 paired NSCLC samples (tumor) and adjacent normal samples (normal) were detected by qRT-PCR. **(B)** TFAP2A levels in NSCLC cell lines were detected by qRT-PCR. **(C)** siRNAs were designed and used to knock down the levels of TFAP2A in NSCLC cell lines. **(D)** CCK-8 assay was conducted in NSCLC cell lines after treatment with si-TFAP2A-1.** (E)** Represent image of colony formation assay of NSCLC cell lines after treatment with si-TFAP2A-1 (left). Colony formation number was quantified by ImageJ software (right). **(E)** Represent image of Transwell assay of NSCLC cell lines after treatment with si-TFAP2A-1 (left). **(F)** Invasive cell number was quantified (right). **P < 0.01.

Next, we explore TFAP2A expressions in NSCLC tissues and cells to find it upregulated as CES3 did (Figure 5A and 5B). Thus, we used siRNAs against TFAP2A in NSCLC to investigate the role of TFAP2A. To further validate that TFAP2A exerts its effects by regulating CES3 expression, we conducted rescue experiments. In these experiments, cells with TFAP2A knockdown were designated as the knockdown group, while CES3 was overexpressed to restore its expression in the rescue group. Figure 5C showed si-TFAP2A-1 notably reduced TFAP2A expression in NSCLC cells. CCK-8 assay showed suppression of TFAP2A inhibited NSCLC growth, and upregulation of CES3 reversed the above impact (Figure 5D). Besides, down regulation of TFAP2A suppressed NSCLC cell colony formation ability, which could be reversed by CES3 overexpression (Figure 5E). Moreover, TFAP2A inhibition suppressed NSCLC cell invasion, which could also be reversed by CES3 overexpression (Figure 5F). All these results demonstrated that TFAP2A inhibition could suppress NSCLC cell growth and invasion.

### TFAP2A inhibition suppresses NSCLC progression via lipid metabolism regulation

**Figure 6:**
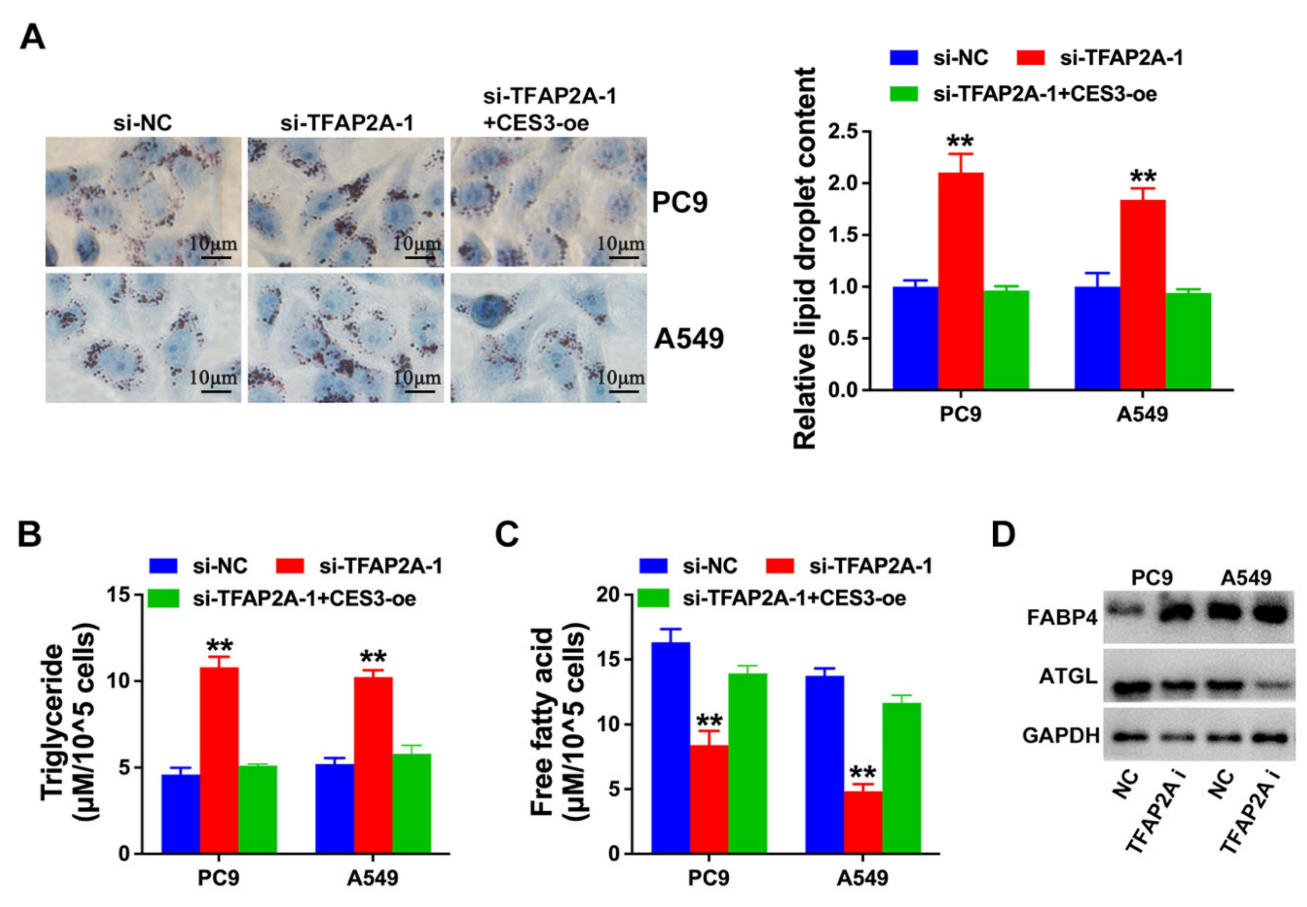
** TFAP2A inhibition suppresses NSCLC progression via lipid metabolism regulation. (A)** Oil red O staining conducted to detect intracellular lipid contents in NSCLC cell lines after treatment with si-TFAP2A-1 (left). Intracellular lipid droplet contents were quantified by ImageJ software (right).** (B)** The intracellular TG levels were detected in NSCLC cell lines after treatment with si-TFAP2A-1. **(C)** The intracellular FFA levels were detected in NSCLC cell lines after treatment with si-TFAP2A-1.** (D)** Lipid metabolism related genes expressions were determined by Western blotting in NSCLC cell lines after treatment with si-TFAP2A-1. **P < 0.01.

Next, we explored the role of TFAP2A and CES3 in NSCLC lipid metabolism. Figure 6A revealed TFAP2A inhibition enhanced NSCLC cell lipid accumulation, which could be reversed by CES3 overexpression. Moreover, TFAP2A inhibition resulted in elevated TG levels and reduced FFA levels in NSCLC cells, which could also be reversed by CES3 overexpression (Figure 6B and 6C). Besides, we found TFAP2A inhibition increased the expression of FABP4, but reduced the level of ATGL in NSCLC (Figure 6D). All these results demonstrated that TFAP2A played vital roles in NSCLC lipid metabolism via regulating CES3.

## Discussion

Significant and revolutionary advances have been made recently in NSCLC treatment, especially in precision medicine and targeted therapy 28-30. Despite improvements in NSCLC screening, diagnosis and treatment, the outlook for some NSCLC patients remains bleak 31, 32. Recurrence and metastasis are important factors affecting NSCLC prognosis, and overall survival rate of advanced NSCLC is low, and its intractability imposes a huge economic burden on the society as well as a great pressure on patients' families. Therefore, exploring new therapeutic approaches for NSCLC is imminent, and we urgently need to seek novel therapeutic targets in NSCLC 33-35. The development of diagnostic and prognostic markers for the early identification and treatment of NSCLC will significantly improve treatment outcomes and further prolong patient survival 36, 37.

Among the many hallmark features of tumors, metabolic remodeling, the process by which tumor cells reconfigure their metabolic networks to remain proliferation, has important regulatory roles in tumor growth and drug resistance 38. Lipid metabolism dysregulation is a significant metabolic change in tumors 39. Lipid metabolism remodeling specifically includes an increased rate of fatty acid production, faster external uptake and transport, increased lipid droplet storage, and altered ratios of fatty acid oxidation to generate ATP. Targeted inhibitors of key genes of lipid metabolism are in full swing in tumor therapy. CES3 have been increasingly recognized as key regulators of lipid metabolism in various physiological and pathological contexts. Studies have shown that CES3 promotes lipolysis, fatty acid oxidation, and browning of white adipocytes, thereby regulating energy balance and adipogenesis 40, 41. Additionally, CES3 expression is reduced in early liver cancer, and its restoration is associated with decreased tumor development, suggesting a protective metabolic role in cancer 21. These findings highlight CES3's multifaceted role in lipid metabolism and suggest its potential implication in cancer biology. Building on this evidence, our study investigates the role of CES3 in NSCLC, exploring how CES3-mediated lipid metabolic pathways may contribute to NSCLC progression and tumor biology.

In this study, RNA next-generation sequencing of 25 paired NSCLC specimens and adjacent normal tissues revealed that CES3 was upregulated in NSCLC. Moreover, qRT-PCR confirmed the elevation of CES3 in NSCLC tissues and cells (Figure 1). CES3 has been reported correlated with lipid metabolism regulation. Inhibiting the function of CES3 might be a promising strategy in cancer treatment. In our study, inhibiting CES3 resulted in suppressed cell proliferation as well as invasion of NSCLC (Figure 2). Besides, inhibiting CES3 enhanced NSCLC cell lipid accumulation (Figure 3), indicating that CES3 was an important player in NSCLC cell proliferation, invasion and lipid metabolism.

Then, we investigated the reason of CES3 upregulation in NSCLC. And we found that TFAP2A was upregulated in NSCLC. TFAP2A is involved in multiple carcinogenesis. In LUAD, TFAP2A was highly expressed and associated with poor prognosis. Zheng *et al.* revealed that TFAP2A promotes metastasis by inducing epithelial-mesenchymal transition (EMT) through transactivation of PSG9 and activation of the TGF-β pathway in LAUD. Xiong *et al.* further demonstrated that TFAP2A binds to the promoter of CTHRC1, upregulating its expression and activating fatty acid metabolism, which promotes migration and invasion of LUAD cells 42. These studies underscore the multifaceted role of TFAP2A in promoting lung adenocarcinoma metastasis through distinct molecular pathways 43. Here, we found TFAP2A could bind to CES3 promoter and regulate CES3 expression in NSCLC (Figure 4). Advances in emerging technologies are revolutionizing the study and treatment of cancer. Approaches including single-cell multi-omics, spatial transcriptomics, and CRISPR-based functional genomics enable unprecedented resolution in dissecting tumor heterogeneity, metabolic networks, and transcriptional regulation *in situ* 44, 45. Application of these state-of-the-art tools to NSCLC models could further clarify the dynamic regulation of CES3 and TFAP2A in the tumor microenvironment and identify biomarkers predictive of response to metabolism-targeted therapies.

Subsequent experiments showed that inhibition of TFAP2A suppressed NSCLC proliferation and invasion, while overexpression of CES3 could reverse the above effects (Figure 5). TFAP2A has been reported associated with cellular metabolic process regulation, indicated by GO analyses 46. In LUAD, TFAP2A could enhance glycolysis via stimulating HMGA1 expression 47. Moreover, TFAP2A could bind to the promoter of several lipid droplet, leading to enhanced lipid droplets biogenesis and accumulation in cells 48. Here, we also found that TFAP2A could regulate NSCLC lipid metabolism via CES3 (Figure 6). Targeting lipid metabolic pathways, including enzymes like CES3, holds promise for cancer therapy but also presents inherent challenges and potential risks. Because lipid metabolism is essential not only for tumor cells but also for normal cellular functions, immune system activity, and whole-body energy balance, disrupting these pathways may lead to adverse effects such as hepatic dysfunction, metabolic imbalances, or impaired immune responses 49. Moreover, cancer cells often display metabolic flexibility, which can enable them to bypass inhibited pathways and limit the long-term effectiveness of such treatments 50-52. Therefore, while CES3 inhibition shows encouraging antitumor effects *in vitro*, it is crucial to thoroughly evaluate its 1safety and systemic impact *in vivo*. Future studies employing relevant animal models—such as genetically engineered mice or patient-derived xenografts of NSCLC—are necessary to validate the therapeutic potential of targeting CES3, assess treatment efficacy, and monitor possible toxicities. This stepwise preclinical validation will be indispensable for translating CES3-targeted strategies into safe and effective clinical interventions.

Despite the significant insights gained from our study, several limitations should be considered. First, the absence of *in vivo* experiments in animal models limits the ability to fully understand the biological relevance and therapeutic potential of targeting the TFAP2A/CES3 axis in a physiological context. Second, although we demonstrated that TFAP2A regulates CES3 expression and influences lipid metabolism in NSCLC cells, the detailed molecular mechanisms by which the TFAP2A/CES3 axis modulates key lipid metabolism regulators such as FABP4 and ATGL remain unclear and warrant further investigation. Elucidating these pathways will enhance understanding of lipid metabolic reprogramming in NSCLC and may identify additional targets for therapeutic intervention. Addressing these limitations will be crucial for translating our findings into effective clinical strategies.

## Conclusion

Here, we revealed that CES3 was elevated in NSCLC and acted as an oncogene in NSCLC progression. And CES3 regulated NSCLC tumorigenesis via regulating lipid metabolism. Mechanically, TFAP2A could regulate CES3 levels in NSCLC, that the dysregulation of TFAP2A resulted in CES3 overexpression and the following NSCLC tumorigenesis. We could use TFAP2A/CES3 as a novel strategy to treat NSCLC in the future.

## Supplementary Material

Supplementary tables.

## Abbreviations

NSCLC: non-small cell lung cancer
CES3: carboxylesterase 3
TFAP2A: transcription factor AP-2α
TG: triglyceride
FFA: free fatty acid
FABP4: fatty acid-binding protein 4
ATGL: adipose triglyceride lipase
ChIP: chromatin immunoprecipitation
LUAD: lung adenocarcinoma
LUSC: lung squamous cell carcinoma
