# Supplementary material for: CES3 promotes NSCLC progression via lipid metabolic reprogramming regulated by TFAP2A
Source: J Cancer. 2026 Jan 1;17(1):99–108. doi: 10.7150/jca.118395 (PMC12719574; doi:10.7150/jca.118395)
Supplement: Supplementary file 1 — Supplementary tables. [file jcav17p0099s1.pdf]

Table S1. The sequences of siRNAs used in this study

Name	Species	Target Sequences
si-NC	Human	UUCUCCGAACGUGUCACGUTT
Si-TFAP2A-1	Human	CCAATGAGCAAGTGACAAGAA
Si-TFAP2A-2	Human	CCCAGATCAAACTGTAATTAA
Si-TFAP2A-3	Human	GCTCCGGGATCAGCAACCCTT

Table S2. Primer sequences for qRT-PCR used in this study

Construct	Species	Direction	Sequence (5' - 3')
CES3	Human	Forward	CCCACCGTCAGTTTTTCAAGA
		Reverse	GGGTCGATGCTGGAACTCATA
TFAP2A	Human	Forward	CTCCGCCATCCCTATTAACAAG
		Reverse	GACCCGGAACTGAACAGAAGA
GAPDH	Human	Forward	AGCCACATCGCTCAGACAC
		Reverse	GCCCAATACGACCAAATCC
